# Reperfusion injury in acute myocardial infarction

**DOI:** 10.1186/cc11280

**Published:** 2012-06-07

**Authors:** Gregor Simonis, Ruth H Strasser, Bernd Ebner

**Affiliations:** 1 Department of Medicine/Cardiology, Heart Center, Dresden University of Technology, Dresden, Germany

## Background

For many years it has been shown that the size of a myocardial infarction is not only determined by ischemic damage, but also by reperfusion itself. This reperfusion injury contributes to up to 50% of the final infarct size. Mechanical postconditioning using short periods of ischemia immediately after reperfusion, pharmacologic postconditioning targeted to prevent opening of the mitochondrial permeability transition pore, and hypothermia have been shown to prevent reperfusion injury in animals and to reduce infarct size in smaller studies. Larger studies targeted to reduce heart failure and mortality are eagerly awaited.

## Introduction

Since the early 1970s, infarct size has been identified as a major predictor of prognosis after myocardial infarction: infarct size parallels with arrhythmia, heart failure, and mortality [1]. Apart from this finding, infarct size is not only determined by the area at risk; that is, the myocardium perfused by the infarct-related artery. In a seminal work, Reimer and coworkers could show that irreversible myocardial injury, as determined by cardiac myocyte necrosis, progresses as a wavefront from the subendocardium towards the subepicardium [2]. This observation led to the concept that early reperfusion therapy can salvage myocardium at risk from injury. Early reperfusion therapy within the first 3 to 6 hours after the onset of ischemia was, in consequence, rapidly introduced into clinical routine and is now standard treatment of patients with acute myocardial infarction.

In parallel, it was recognized that reperfusion of temporarily nonperfused myocardium itself has effects on cellular integrity [2]. Reperfusion saves viable myocytes. However, it accelerates the disruption of irreversibly injured myocytes, and thereby permits the process of inflammation, phagocytosis, and infarct repair to begin quickly. It leads to interstitial hemorrhage from vessels that are injured by ischemia but are reperfusable at the time of reflow; that is, in the border zone of the infarction. The hemorrhage itself increases the interstitial pressure, which in consequence worsens the tissue perfusion again. Moreover, reperfusion induces severe morphologic alterations of the myocardium such as cardiac myocytes swelling, mitochondrial damage, hypercontracture, and loss of myofibrillar organization [3]. The observation that reperfusion itself influences infarct size has led to the concept of lethal reperfusion injury [2,3]. It became quickly clear that reperfusion injury is not only determined by mechanical factors such as hemorrhage or interstitial pressure. Reperfusion leads to the activation of many signaling pathways that contribute independently to both apoptotic and necrotic tissue injury and thus decrease the amount of viable myocardium (reviewed in [4,5]). The concept of additional myocardial damage is induced by lethal reperfusion injury has been supported by the observation that interventions started before reperfusion can reduce infarct size, as discussed below [4,5]. Studies in animals suggest that lethal reperfusion injury accounts to up to 50% of the final size of a myocardial infarction.

Clinically, reperfusion injury may be seen in four different types of cardiac dysfunction: myocardial stunning - that is persistent mechanical dysfunction despite restored blood flow which is usually reversible within weeks; the no-reflow phenomenon after opening of an infarcted coronary artery; reperfusion arrhythmia; and lethal, irreversible injury of the myocardium. In the recent years, rapid revascularization was instituted to prevent reperfusion injury. From a clinical view, currently there seems to be no potential to further reduce infarct size by faster restoration of blood flow. Therefore other than mechanical strategies to reduce reperfusion injury and in consequence infarct size are highly welcome to improve the outcome of the patients. To further understand potential strategies, the molecular mechanisms contributing to reperfusion injury are of importance.

## Signals contributing to reperfusion injury

The signals contributing to reperfusion injury have been recently reviewed in detail [5]. Major contributors are as follows.

### Oxidative stress [6]

which is discussed as the oxygen paradox since the reoxygenation of ischemia generates a myocardial injury that exceeds the injury of ischemia alone. Oxidative stress diminishes the cardioprotective effects of nitric oxide, leading to more neutrophil activation, increased levels of superoxide radicals, and diminished myocardial blood flow.

### Increased intracellular calcium

occurring secondary to the ischemic damage of the sarcolemmal membranes and to the oxidative stress-induced dysfunction of the sarcoplasmic reticulum [7]. The calcium excess induces cardiac myocytes death by hypercontracture and opening of the mitochondrial permeability transition pore (mPTP), a molecule recently identified as one of the most important targets in reperfusion injury [8].

### *The rapid restoration of physiologic pH *during reperfusion

which follows the washout of lactic acid. This leads to the activation of the sodium-hydrogen exchanger, finally leading to mPTP opening [9].

### Inflammation

leading to neutrophil accumulation and their transmigration into the myocardial tissue. These neutrophils cause vascular plugging and release degradative enzymes and reactive oxygen species [10].

In addition to these more traditional factors, the reperfusion injury salvage kinase (RISK) pathway and its effector, the mPTP, have been currently postulated to be centrally involved in reperfusion injury [8,11,12]. Opening of the mPTP, which releases calcium from the mitochondria and leads to intracellular calcium overload, does not occur in ischemia but is a key determinant of the first few minutes of myocardial reperfusion. Some authors discuss that this pathway could serve as the final effector of the above discussed contributors to reperfusion injury.

## Treatment in humans

Whereas in animal studies many agents were successful to reduce reperfusion injury, the translation of these results into the clinical setting has been disappointing for many years [5]. Several groups tried to target oxidative stress with antioxidants or nitric oxide supplementation. These trials had negative results [5,13,14]. Comparably, trials of inhibition of the sodium-hydrogen exchanger, which have been successful in animals by reducing the intracellular calcium overload in reperfusion and by delaying pH normalization in the reperfused myocardium, failed in humans [15], as have various measures to reduce the inflammatory damage induced by neutrophils [5]. Trials with metabolic modulation such as glucose-insulin-potassium infusions or magnesium therapy had inconclusive results. Together, these measures addressing the traditionally seen contributors to reperfusion injury were, at least in humans, not successful to clearly reduce myocardial damage.

Some attempts have clinically been done to treat the no-reflow phenomenon. The administration of platelet glycoprotein GP IIb/IIIa blockers or thrombus aspiration improves reflow after vessel reopening, with limited influence of infarct size. Adenosine is widely used to treat slow-flow phenomena in the infarct-related artery. Of note, the administration of adenosine as an anti-inflammatory and vasodilatatory agent during reperfusion could reduce infarct size in humans, making adenosine currently the only clinically used drug which improves both reflow and infarct size. Adenosine, however, did not reduce clinical end points, which was the primary end point of this study [16].

Three strategies are currently discussed as innovative treatment modalities for reperfusion injury in acute myocardial infarction: mechanical postconditioning, pharmacological postconditioning with substances influencing the RISK pathway and the mPTP, and hypothermia.

### Mechanical postconditioning

In 2003, Zhao and coworkers could demonstrate that short periods of ischemia and reperfusion applied immediately after reopening of an infarct-related artery could reduce reperfusion injury and infarct size, a concept referred to as ischemic postconditioning [17]. In animal studies, ischemic postconditioning involves all major contributors to reperfusion injury including the RISK pathway and the mPTP. Various smaller clinical studies showed in humans that repetitive inflations of an angioplasty balloon after reopening of the infarct-related artery reduces infarct size, wall-motion scores and comparable end points [18-20]. A related strategy, which has also shown to be effective in animals and humans, is the remote ischemic postconditioning. Here, transient episodes of ischemia and reperfusion in a remote organ - that is, skeletal muscle - protect the heart from reperfusion injury. A very elegant randomized trial could show that the simple, non-invasive, repetitive inflation of a standard blood pressure cuff in patients with acute myocardial infarction reduces infarct size when applied before the reopening of the infarct-related artery [21]. Given the confirmation of this finding in larger trials, mechanical, remote postconditioning could be an easy and safe measure to reduce reperfusion injury.

### Pharmacologic postconditioning

Extensive preclinical evidence showed that pharmacologic activation of the RISK pathway or prevention of mPTP opening reduces reperfusion injury. Some drugs addressing the RISK pathway have been tested in proof-of-concept studies in patients. Of those, inhibition of the protein kinase C-isoform delta with the substance KAI-9803 given into the infarct-related area immediately before reperfusion [22] and administration of high-dose atorvastatin before PCI [23] are the most promising, both showing favorable effects in about 150 randomized patients. Another clinical trial addressed directly the mPTP. In a small pilot trial including 58 patients, the i.v. administration of cyclosporine (2.5 mg/kg), known to inhibit mPTP opening, reduced infarct size and the release of cardiac markers [24]. Due to the limited numbers of patients, however, it is not clear whether those interventions result in the reduction of clinical endpoints.

### Hypothermia

For a couple of years, it has been known that in animals hypothermia instituted before the reopening of an infarct-related coronary artery reduces reperfusion injury and thus infarct size [25]. For infarct size reduction, hypothermia has to be instituted before reperfusion [26], whereas cooling directly after vessel reopening is unsuccessful in most cases [26]. It is discussed that hypothermia prevents the reactive hyperemia in early reperfusion.

Few studies examined the effects of hypothermia in patients with acute myocardial infarction. Dixon and colleagues could show that therapeutic hypothermia is safe in myocardial infarction patients, however, without influencing outcomes [27]. Wolfrum and colleagues showed in patients with myocardial infarction subjected to CPR that institution of therapeutic hypothermia before revascularization did not prolong door-to-balloon times [28]. Two yet unpublished studies, the COOL-MI study and the ICE-IT study, showed no effect on infarct size in the total population. However, in patients with anterior myocardial infarction cooled to <35°C at the time of reperfusion, infarct sizes were roughly halved [29]. The treatment of awake patients not subject to CPR is, however, limited due to counter-regulatory processes such as shivering, which leads to increased oxygen demand and workload of the heart. Future studies will test hypothermia in patients with myocardial infarction, using a straightforward adjunctive treatment with buspirone and meperidine to prevent shivering. In this setting, clear treatment protocols are necessary to avoid prolongation of the door-to-balloon time in patients with myocardial infarction.

## Problems still to face

### Signaling

Some conditions have been identified to inhibit salvage from reperfusion damage like myocardial hypertrophy, diabetes, age, and hypercholesterolemia (reviewed in [30]). All these conditions are associated with limited ability to successfully activate the RISK pathway. Either they increase the threshold of mechanical postconditioning or they abolish myocardial salvage at all. As these conditions are highly present in the patient population, further research may be of relevance to reestablish susceptibility to modified reperfusion strategies.

### Timing

All proposed strategies to prevent reperfusion damage assume a relevant amount of reperfusable myocardium that is waiting to be salvaged. All planned measures to reduce reperfusion damage before revascularization should preferably be applied in a very short time.

### Infarct size

Hard clinical endpoints can only be met when there is a significant clinical benefit to be achieved. Posterior infarcts or smaller infarcts of the anterior region only affecting small or medium-sized vessels do not go along with high mortality and therefore there are not many lives to be saved by an optimal reperfusion strategy.

## Conclusion

Reperfusion injury contributes to up to 50% of the total myocardial damage. In spite of many successful results in animals, the translation into the clinical setting has been disappointing for many years. Recently, mechanical, remote ischemic postconditioning as well as pharmacologic postconditioning with substances addressing the RISK pathway and the mPTP have shown positive effects on surrogate end points in patients. Hypothermia may be another option in the treatment of reperfusion injury. Larger studies exploring the potential of these therapeutic options to influence clinically relevant endpoints are eagerly awaited. Currently, the evidence is insufficient to permit a widespread clinical use of those interventions.
